# Linking personal initiative and family help as well as social support: a case study of learning challenges and solutions for older adults in rural China during the COVID-19 pandemic

**DOI:** 10.3389/fpubh.2024.1384527

**Published:** 2024-10-09

**Authors:** Hao Cheng

**Affiliations:** School of Education, Central China Normal University, Wuhan, China

**Keywords:** the COVID-19 pandemic, aging population, educational gerontology, self-directed learning, intergenerational learning, online education

## Abstract

**Background:**

The sudden outbreak of the COVID-19 Pandemic has caused serious damage to the continuous learning of older adults around the world. While the existing literature focused more on older adults’ learning in developed countries, few studies explored older adults’ learning in developing countries with low social and cultural capital.

**Methods:**

This study took family-school cooperation in China as the platform and explored learning challenges and solutions through unstructured interviews with 12 older adults.

**Results:**

The study found that due to policies of working and studying from home, older adults face obstacles in accessing physical learning institutions and digital learning knowledge, skills, and psychology. I further found that the older adults were not forced to accept the challenge passively, but created a lifelong learning model with Chinese characteristics by building self-learning based on personal initiative, developing sustainable intergenerational learning rooted in Chinese family culture, and participating in online learning in schools and enterprises under the government guidance.

**Conclusion:**

This study provided new knowledge for understanding the learning challenges and solutions of older adults in rural China. It is emphasized that policy value and practice enlightenment were highlighted and discussed in conversations with active aging, intergenerational learning, and harmonious societies.

## 1 Introduction

Currently, the proportion of older adults in the global population is significantly increasing (1). The changes in population structure are caused by the decline in global birth rates and improvements in healthcare and living conditions. According to United Nations data, the population aged 60 and above is expected to double to approximately 2.1 billion by 2050 (2). The acceleration of global population aging has brought special challenges, such as changes in disease prevalence, increased medical expenses, scarcity of labor force, utilization of educational resources, and potential issues with retirement income security (3). To address the challenges of global population aging, several studies showed the need for a comprehensive and proactive approach to meet the needs and well-being of older adults (4–7). Collaboration between the government, healthcare system, community, and individuals was crucial for successfully addressing this demographic shift (8).

Several studies demonstrated that education and learning for older adults were crucial, efficient, and beneficial in addressing population aging (9–12). However, all aspects of society, such as the economy, politics, culture, ecology, and education for older adults, were seriously affected by the COVID-19 pandemic (13). Due to school closures, difficulties in accessing educational resources and hindering preferred learning methods were major challenges (14). It is crucial to find effective solutions to ensure that older adults have access to quality education and learning opportunities during the pandemic. Nevertheless, there is a lack of extensive discussion in the academic community on this key research field. Although some studies investigated the impact of COVID-19 on older adults’ education, learning, and life, and proposed solutions to provide education for older adults in nursing homes and other institutions (15–17), these measures were mostly implemented in developed countries with appropriate social welfare, but may not be feasible in developing countries that use emerging information technology. Unfortunately, the current research on how developing countries respond to the challenge and experience of education for older adults caused by the COVID-19 pandemic has not been involved. It needs to be recognized that education solutions must be tailored to the unique challenges faced by various countries for older adults.

With the rapid aging of the population, China is undergoing a major demographic transformation. According to the seventh national population census data released in 2021, the population aged 60 and above reached 264 million, accounting for 18.7% of the total population (18). Various departments, including older adult education, are facing the challenge of population aging. The COVID-19 pandemic has intensified the educational challenges faced by older adults (19). The COVID-19 pandemic has led to nationwide lockdowns, the closure of educational institutions, and restrictions on social gatherings, which have seriously disrupted the traditional education model for older adults (20). Due to the need to adapt to distance learning or complete lack of access to educational resources, educational inequality among older adults has intensified (21). However, China is striving to address the challenges posed by the COVID-19 pandemic and ensure that older adults can continue to receive education through a combination of online learning platforms, offline activities, and community participation (22). In fact, during COVID-19, China has made effective progress in addressing the educational needs of older adults (23). The experience gained in this process helps to realize the educational rights and learning needs of older adults. Unfortunately, these relatively successful and beneficial experiences have not been widely disseminated through academic research. The insufficient dissemination of these experiences hinders the practical progress and theoretical exploration of global education for older adults. Therefore, this study aims to explore the learning challenges and potential solutions faced by older adults in China during the COVID-19 pandemic. The value of this study can be observed from two different perspectives. Firstly, it provides new knowledge on older adults’ education in China, which has certain theoretical value, thus contributing to the establishment of a diversified older adults’ education system worldwide. Secondly, it provides specific enlightenment for policy formulation, especially for countries with similar national conditions to China to cope with the challenges brought by the COVID-19 pandemic.

The introduction provided a global perspective on the challenges faced by older adults during the COVID-19 pandemic. The literature review critically analyzed the previous studies on the challenges and potential solutions for older adult learners to cope with the COVID-19 pandemic and assessed the progress and limitations. The methodology introduced China’s education policies for older adult learners during the COVID-19 pandemic and the in-depth interviews with 12 participants. The research results showed how personal initiative, family intergenerational learning, and social support can help to enhance the ability of older adults to overcome the learning challenges brought by the COVID-19 pandemic. The conclusion and discussion emphasized the theoretical and practical significance of the research while acknowledging potential limitations. In addition, this section outlined the prospects for further investigation in the context of research findings.

## 2 Literature review

Overall, the perception and potential impact on older adults, especially those who are vulnerable and multifaceted. Among them, scholars were most concerned about the significant physical and psychological impact of the COVID-19 pandemic on older adults (24, 25). The COVID-19 pandemic has replaced the face-to-face activities of older adults with online activities, severely limiting their physical activities. A study on older adults in Australia found that although online exercise helped improve physical health, alleviate depression, and life quality, its effect was far less than outdoor exercise before the COVID-19 epidemic (26). Another eight-week online aerobic dance program held at home showed that online exercise programs were feasible, fun, and safe, providing strong support for older adults who were unable to engage in outdoor exercise (27). In terms of psychological impact, the sudden COVID-19 pandemic has brought older adults from the real world into virtual life, and strangeness, discomfort, and fear have become the daily psychological reflections of many older adults. For example, a survey of 105 older adults in Israel found that not all older adults benefit from online activities such as interpersonal communication and alleviating loneliness, and there were a large number of negative mental health issues. Older adults exhibited tension and fear when facing information technology (28). Another example, a survey involving 315 older adults in Spain showed that the COVID-19 pandemic led to severe age discrimination against older adults, and anxiety, pressure, and fear became the main psychological state of most older adults (29). In general, these studies, which received much attention from scholars, were aimed at exploring the impact of the COVID-19 pandemic on the physical and mental health of older adults (30). Unfortunately, these researchers have not yet explored some approaches to improve the mental health of the older adults, such as education or learning.

Globally, it is generally believed that older adults who follow the principles of continuous education and lifelong learning are more likely to achieve active, successful, healthy, and productive aging than those who do not participate in learning activities in their daily lives (31–34). In response, some studies explored the provision of education and learning support to older adults who faced obstacles to the right to education and learning opportunities during the COVID-19 pandemic (35–37), to help older adults cope with unexpected crises that may occur in their daily lives. It is worth noting that most of the studies on older adults learning during the COVID-19 pandemic were conducted in school educational institutions or nursing homes. A study pointed out that the COVID-19 pandemic had a profound impact on older adults learning. Fortunately, public health nursing education outside the family provides an important way for older adults to participate in learning (15). Another study in South Florida also highlighted the positive value of social and educational programs for older adults during the COVID-19 pandemic crisis (38). In fact, many studies emphasized the value of social organizations or institutions such as schools and learning centers for older adults, enabling them to access education and learning opportunities (39). It should be pointed out that most of these studies were conducted in developed countries with high welfare societies, where a developed social economy and comprehensive public services can provide important support for the education and learning of older adults. However, some developing countries may not be able to access these supportive measures aimed at safeguarding the learning rights and opportunities of older adults. For example, in China, where the population is rapidly aging, social public services face challenges in meeting the educational needs of each older adult within a limited time frame (40, 41). Therefore, family and community were expected to become one of the effective ways for older adults to obtain lifelong education and practice lifelong learning (42). However, the education and learning experiences of developing countries need to be further developed and disseminated to enhance our understanding of the diverse educational models for older adults.

Cultural capital, which is composed of physical health, education level, economic level, and professional characteristics, is an important indicator to measure individual social status and has become the premise of almost all social science research (43). This general viewpoint has also been confirmed in many studies on lifelong education and older adults’ learning (44, 45). In addition, older adults with higher social and cultural capital are more likely to obtain relatively high-quality educational resources and learning methods (46), while older adults with lower social and cultural capital tend to lose opportunities for education and learning due to physical and mental health problems (47). In the post-modern society with outstanding achievements, older adults with high social and cultural capital are often seen, while older adults with low social and cultural capital are often ignored (48). This phenomenon is also reflected in the research on how to provide educational support for older adult learners during the COVID-19 epidemic. For example, in the context of big data society, some studies specifically discussed the advantages and convenience of urban older adults in access to education resources (49, 50), but had not paid attention to the challenges and solutions of rural older adults in access to education rights. In addition, other studies also made similar or even consistent claims (15, 51). Older adults living in rural areas often find it difficult to obtain lifelong education and learning support and assistance, and education and learning have almost become a priority for healthy older adults in urban (10). To change this situation, educational researchers proposed to broaden the research perspective and include older adults with low social and cultural capital, aiming to break the monopoly of older adults with high social and cultural capital on lifelong education and learning (52–54). However, it is regrettable that the current research on providing educational resources and learning opportunities for vulnerable older adults in rural areas during the COVID-19 epidemic is still insufficient. It will undoubtedly become an important force restricting the current theoretical construction and practical promotion of global older adult education.

## 3 Methodology

This study began with the impact of the COVID-19 epidemic on the educational rights and learning opportunities of rural older adults with low social and cultural capital. This research question dates back to December 2019, when multiple cases of unexplained pneumonia were detected one after another in Wuhan, China. On February 11, 2020, the World Health Organization named this respiratory infection with acute features ‘Corona Virus Disease 2019’ and highlighted its potential to become a global pandemic (55). In the face of the sudden outbreak of the COVID-19 epidemic, the Chinese government adhered to the people-centered concept of governing the country and led Chinese people of all ethnic groups to fight against the social crisis (56). This situation tests both China’s awareness and ability to cope with social crises during special times and its achievement in developing a modern socialist system with unique features.

To better outline and understand education policies and practices for older adults in response to the COVID-19 epidemic, it may be necessary to explain China’s administrative division. China’s administrative structure is a six-level unit comprising the Nation, Province, Municipality, County, Township, and Village (57). China’s policies and regulations are predominantly implemented through top-down transmission (58). The Chinese government implemented various policies to mitigate the adverse effects of the COVID-19 epidemic on education for older adults. For example, on January 27, 2020, the Ministry of Education issued the *Notice on Postponement of the spring Semester 2020*, requiring local educational administrations at all levels to make arrangements for postponing the start of the semester and suggesting that the older adult schools provide support for the older adults to study at home (59). As another example, on March 6, 2020, the Ministry of Civil Affairs issued the *Notice on Strengthening Care Services for Older Adults with Special Difficulties during the Epidemic Prevention and Control Period*, calling on social work organizations, psychological counseling organizations, and other professional forces to provide timely educational support, psychological comfort, and other services for older adults with special difficulties in need through a combination of online and offline methods such as network counseling hotlines and door-to-door services (60). Since achieving uniformity between policy formulation and implementation is challenging, implementing these policies may be difficult. Nonetheless, these policies provide crucial guidance and direction for vulnerable older adults in rural China to acquire educational and learning support during the COVID-19 pandemic.

This study was conducted in Wuyi County, Jinhua Municipality, Zhejiang Province, China. Covering a total area of 1,577 square kilometers, Wuyi County is located between latitudes 28°31′–29°03’N and longitudes 119°27′–119°58′E. The selection of this study case was based on two factors. First, I established long-term cooperative partnerships with educational administrative departments, community schools, primary and secondary schools, as well as principals, teachers, students, and parents in Wuyi County. These partnerships focus on integrating educational theory and practice, fostering rural home and school cooperation, and promoting intergenerational learning. This strong collaboration provided a solid practical foundation and a conducive environment for conducting this study. Second, the initial investigation revealed that, despite the challenges posed by the COVID-19 pandemic, the vulnerable rural older adults in this region had access to lifelong education and learning. This observation is an appropriate response to the research questions we formulated.

From June to October 2020, I conducted a research on intergenerational learning to help students’ grandparents bridge the digital divide in collaboration with a classroom teacher Shuli (pseudonym) at Quanxi Primary School in Wuyi County. Shuli, a 48-year-old woman with an outgoing personality, was good at telling me about her practical experiences in classroom construction, home and school cooperation, intergenerational learning, and other program research. Along with the continuous and in-depth development of this study, I established a long and solid relationship with Shuli in terms of educational theory research and educational practice change. Considering the convenience and practicability of conducting the research, I still wanted to collaborate with Shuli to investigate the learning difficulties and solutions experienced by rural older adults who were disadvantaged during the COVID-19 pandemic. In January 2021, I contacted Shuli via WeChat, an online social networking platform, to explain the value of this study, and wanted to work with her on this study from an international perspective. I was pleased to find that Shuli was enthusiastic and willing to participate in this study as a practitioner. With the strong support and assistance of Shuli, I initiated the process from research design to implementation. Note that there were 36 students in her class, 29 of whom were from rural with relatively low social and cultural capital. Deeply influenced by Confucianism, the living together of grandparents, children, and grandchildren has become the dominant cultural practice in Chinese families (61). Twenty-three students from rural with low social and cultural capital lived with three generations. The grandparents of the 23 students were invited to participate in this study to explore the learning challenges and solutions experienced during the COVID-19 pandemic with the support of Shuli. Adhering to the principle of voluntariness and non-coercion, I recruited 12 older adults from 12 families to participate in this study. The demographic information of them is below (Table 1).

**Table 1:** Participants’ demographic information.

Pseudonym	Gender	Age	Education background
Ling Qing	Female	71	Primary school
Hong Xiao	Female	66	Junior high school
Min Wei	Male	64	Senior high school
Xiu Yu	Female	70	Primary school
Zhu Liang	Male	62	Primary school
Hui Ze	Female	63	Primary school
Yi Peng	Male	65	Junior high school
Xia Qing	Female	73	Junior high school
Long Yun	Male	67	Senior high school
Qing Guo	Male	66	Primary school
Jin Qiu	Female	66	Junior high school
Ning Xiang	Female	70	Primary school

To obtain more unanticipated results, I chose in-depth interviews as a data collection method in comparison to questionnaires (62). At the time of data collection, China had not yet implemented a policy of complete freedom of travel due to the uncertain global outbreak of the COVID-19 pandemic. To facilitate the research process as planned, I conducted interviews with older adults by calling and using WeChat online. Before the interviews were formally conducted, I informed 12 older adults about the purpose, timing, and confidentiality of the interviews to encourage the participants to freely express their learning challenges and potential solutions during the COVID-19 pandemic. The interview consisted of two questions. First, what are the challenges of the COVID-19 pandemic to your learning? Second, how did you persist in continuing your studies in the face of these challenges and what help did you receive in seeking out education resources and learning ways? Each older adult participated in an interview lasting between 40 and 60 min. Four older adult participants were interviewed two to three times due to temporary disruptions and suboptimal internet connections.

Audio recordings of interviews were transcribed verbatim, then translated into English and thoroughly checked and verified for accuracy. The interview data were analyzed using thematic coding (63, 64). By using the interviewed older adults’ linguistic expressions to present the challenges that the COVID-19 pandemic posed to their learning, as well as to illustrate what they looked for to help them successfully navigate the social crisis. To ensure consistency in coding, two colleagues with similar research interests to mine were invited to participate in coding the interview data in addition to me. After a small amount of tweaking and modification, a more consistent consensus was reached between the coding of my two colleagues and myself (65). By comparing and adjusting for consistency, the subjective biases of the coders were eliminated to the greatest extent possible (66).

## 4 Findings

### 4.1 COVID-19 presents two learning challenges for older adults

#### 4.1.1 Blocking older adults from going to educational institutions outside their home

As the concepts and actions of lifelong education, lifelong learning, and learning society have been widely disseminated globally, the Chinese government formulated a development strategy to establish an education system that encompasses lifelong learning for all (67). It is noteworthy that the growth of education for older adults has become an essential element of this development strategy and system construction. Nowadays, the practical development of education for older adults in China has begun to take shape (68), including various official institutions such as universities for older adults, community schools, open universities, and older adults’ rural learning centers as well as private social activities such as nursing homes. However, the strong momentum of education for older adults in China was suddenly interrupted by the COVID-19 pandemic. As the older adults interviewed expressed, the policy of working, and learning at home caused by the COVID-19 pandemic completely prevented them from going to various physical education centers outside their homes.

*Due to the COVID-19 pandemic, I had to stay at home at the call of the government. Our rural older adult learning centers were closed, which had a particularly negative impact on me who wanted to learn every day. Now, I am looking forward to opening the rural older adult learning centers as soon as possible so that we can return to the previous life of free learning (Jin Qiu).*

*I think the biggest impact of the COVID-19 pandemic on me is isolation from society. I have never experienced such a long time being confined to home. As a lifelong learner, the COVID-19 pandemic made it impossible for me to leave home to go to an educational institution for older adults. I feel uneasy about this (Hui Ze).*

While complaining that they could not go to school for older adults due to the COVID-19 pandemic, the interviewees were also full of memories and nostalgia for their happy learning life in educational institutions before the COVID-19 pandemic. They said that before the COVID-19 pandemic, they could freely enter and leave educational institutions at any time, and they could choose learning content suitable for their own life needs. For them, this is their beautiful expectation for the relationship between education and life. It vividly explains their outlook on life, values, and worldview of learning in life and living in learning.

*I miss those happy days when I could go to the advanced learning center at any time. I can not only learn knowledge there but also make friends. To be honest, before the COVID-19 pandemic, I went to the older adults learning center and had a good time. I do not know when I can return to a life full of learning again (Hong Xiao).*

*Although I can now study online at home, I still enjoy the learning time when I go out of my home to the older adult learning center. However, I am sorry to say that there is currently no face-to-face learning atmosphere with other older adult students in the classroom. I look forward to the end of the COVID-19 pandemic as soon as possible and everything will return to normal (Long Yun).*

It can be seen from the older adults interviewed above that the sudden outbreak of the COVID-19 pandemic has disrupted their normal study and life of leaving their families and entering society. They cannot participate in institutionalized education and non-institutionalized social learning. The once accessible educational venues, resources, and educational models become a luxury, even an impossible dream. Moreover, unfortunately, this impact is often irreparable and immeasurable.

#### 4.1.2 Leading to the internet digital learning barriers for older adults

Accompanied by the iterative upgrading of productive forces and production relations, human society has completed the historical stage of transition from an industrial society to an information society (69). Networking, digitalization, and technicalization, as important features of the current artificial intelligence society, are being integrated into people’s daily life, work, and study at an unprecedented speed (70). However, not all of us can successfully adapt to the current era of rapid development and uncertainty. The younger generation of digital natives is benefiting from their upbringing, enabling them to take advantage of modern technological advances. In contrast, the older generation born between 1940 and 1960 is at risk of being left behind by society due to the generation gap (71). As some studies argued, the digital divide has become a great challenge for many older adults in the current information technology era, especially those with low social and cultural capital (72–74). The challenge of such uncertainty is reflected in education and learning as digital learning disabilities. The resulting theoretical assumptions and extrapolations are occurring silently in the daily life practices of older adults. According to the personal experience of older adults interviewed above, a digital learning disability has become another huge challenge to their learning brought about by the COVID-19 pandemic. Digital knowledge, digital skills, and digital psychology are three important aspects of digital learning disabilities among older adults.

As a representative of older adults who had only attended school, Zhu Liang was lacking in digital knowledge. Like many of the other older adults interviewed, he had little access to electronic devices other than a cell phone before the emergence of the COVID-19 pandemic. As he expressed:

*I have a phone, but I mainly make phone calls and text messages and rarely use any other functions. Although the service staff at the older adult learning activity center provided me with a guide on how to use my phone for online classes, I do not have basic digital knowledge. It is no exaggeration to say that compared to young people, I am digitally illiterate.*

Yi Peng, who has a middle school education, has basic literacy skills, as Zhu Liang calls himself ‘digitally learning disabled’. However, unlike Zhu Liang who tends to emphasize an extreme lack of digital knowledge, he stresses that his deficiency is in the use of digital skills. He was often unsure how to use his cell phone or tablet to access the learning resources recommended by the school for older adults every day. As a result, he could do nothing but laugh bitterly and helplessly in the face of the older adult school’s shift of learning activities from offline to online.

*According to the national recommendation on learning from home, schools for the older adults have arranged learning activities on a specific electronic learning platform. Previously, I had hardly participated in online courses or received training on digital skills, and did not know the operational skills for accessing learning resources. Therefore, I have repeatedly failed to participate in online learning activities arranged by the school for the older adults.*

Contrary to the superficial characteristics of digital knowledge and skills, digital psychology is another important aspect of the COVID-19 pandemic that hinders the digital learning of older adults. Digital psychology refers to the overall psychological state of individuals when facing electronic digital devices and information technology society (75). From the expression of the interviewed older adults, nervousness, confusion, anxiety, irritability, and fear become their psychological reactions to digital Learning disability. An important reason for these negative psychological states is the contradiction between the non-digital life processes of older adults and today’s society characterized by information technology. These difficult-to eliminate negative psychological effects further lead to the loss of confidence in learning among older adults, a decrease in learning expectations, and ultimately, extremely low learning efficiency. As one older adult interviewed helplessly stated:

*The COVID-19 pandemic has completely exposed my shortcomings in digital learning, especially in digital psychology. Due to the difficulty in acquiring digital knowledge and skills, I have been psychologically refusing or somewhat resisting mobile phone-based digital devices. As a pathogenic factor, the COVID-19 pandemic has further aggravated my negative psychology toward digital learning. Nowadays, when digital learning activities are organized in the activity centers of older adults, I unconsciously feel annoyed and afraid, and even a bit dislike learning (Ling Qing).*

Compared to the younger generation, these interviewed older adults have rich life experiences. In the current era of digital transformation, the COVID-19 pandemic has accelerated the transformation of people’s life, learning, and work from offline to online. This unprecedented change has led to difficulties for older adults, especially in rural with low social and cultural capital, in living, learning, and working. As a research institute commented, the comprehensive and rapid digital transformation is grossly unfair and unjust to older adults (76). Creating an inclusive society where both digital and non-digital coexist is one of the essential features of humanity’s journey toward a more civilized society.

### 4.2 Older adults learning at home in the connection of inner salvation and outer help

Faced with the difficulties and challenges of not being able to provide educational institutions for older adults outside of the home, as well as the obstacles of online digital learning, the older adults participating in this study not only passively choose to wait for educational resources and learning opportunities. As a matter of fact, they have created self-directed learning, intergenerational learning, and community online learning through internal redemption and external assistance based on personal, family, and social relationships. In addition, practice showed that these three types of learning are effective in supporting and helping older adults to successfully cope with the learning challenges brought by the COVID-19 pandemic.

#### 4.2.1 Constructing a self-directed learning approach for older adults based on their subjective initiative

The complex and multifaceted relationship between education and self-directed learning has become one of the major topics of current academic discussions (77). From the perspective of whether it is institutionalized or not, learning can be broadly classified into institutionalized and non-institutionalized learning (78). Self-directed learning is a process by which individuals acquire knowledge and skills independently without formal instruction or guidance from teachers (79). As an important component of non-institutionalized education, self-directed learning has increasingly been highly emphasized and recognized in the development of de-schooling thought and practice. Many studies even flagged and distinctly put forward the educational assertion of homeschooling and learning at home, and argued that self-directed learning played an important role in promoting personal and professional development, adaptability, and lifelong learning skills (80).

Faced with the uncertain development of the COVID-19 pandemic, older adults interviewed in this study tended to develop self-directed learning suitable for staying at home according to their living needs and access to educational resources. The way of learning from the subjective initiative was jokingly referred to as ‘self-redemption of learning’. Overall, these older adults had different ways of self-directed learning due to their educational level and life history.

Min Wei, like most of the interviewees, is an older adult who particularly enjoys watching television. He often shared the news and knowledge he learned from TV with friends. Since the COVID-19 pandemic, many TV channels have been broadcasting information about global governance. Getting things related to the outbreak from TV became one of Min Wei’s most important methods of self-directed learning. According to his description, he learned how to wear a mask effectively, clean hygiene properly, and increase physical activity from daily news broadcasts, health programs, and sports channels. In the opinion of many of the older adults interviewed, television as a modern communication medium was able to provide important support for them to learn uninterruptedly about the prevention and treatment of the COVID-19 pandemic. For example, he stated that:

*Before the COVID-19 pandemic, taking care of my grandchildren, watching TV, and going to a learning center constituted my life. Nowadays, under the influence of working and studying at home, my grandchildren are taken care of by their parents. Besides completing learning activities online assigned by the school for older adults, I maintain lifelong learning through television. My friends also offer similar experiences.*

Like Min Wei and other older adults, Qing Guo also liked self-directed learning, but he tended to use smartphones and tablets. For him, although learning through the TV was more authoritative, learning time was relatively fixed, and learning content was preset. Conversely, the greatest advantage of self-directed learning through smartphones and tablets is that learning time and content depend entirely on the learner’s self-arrangement (81). Consequently, older adults who shared the same self-directed learning method with Qing Guo highly evaluated their self-directed learning, which they considered to be a more free, open, and inclusive way of learning.

*Nowadays is no longer the past traditional society, the current society is full of digital information technology. Cell phones and computers have become the state of daily life for almost everyone. Through cell phones and tablets, I have learned a lot of contemporary knowledge, skills, and thinking about aerospace, waste classification, and online shopping. Perhaps it can be said that the COVID-19 pandemic has driven my self-directed learning consciousness and actions.*

It should be emphasized that older adults interviewed not only engaged in self-directed learning through digital devices such as televisions, mobile phones, and computers but also textbooks developed by the school for them. Enhancing the ability to redeem oneself has become a possibility for older adults to cope with social risks and crises.

#### 4.2.2 Sustaining intergenerational learning grounded in Chinese family culture

The family has both physical and emotional significance in China’s social division of labor and state structure. The knowledge and customs constantly generated by the family are called family culture, which is the essence of traditional Chinese culture and the foundation on which society is built (82). While family members in China are connected by blood ties, they also develop emotional relationships in the ethical and moral sense. Among them, “raising” and “filial piety” are the most important pair of family relationships (83). “Raising” implies that the older generation provides the greatest material and spiritual help to the offspring to support their growth based on legal responsibilities and obligations (84). In this regard, some studies vividly referred to the Chinese older generation as “loyal employees” and emphasized that raising offspring is an unconditional physical and psychological contract from the older to the younger (85). In contrast, “filial piety” is the legal responsibility and obligation of the young generation to provide what support they can for the older generation (86). Based on this clarification of the relationship between “raising” and “filial piety,” the older and younger of the Chinese family construct an obligatory reciprocal relationship based on physical, emotional, and legal connections. The study of intergenerational learning in China, which is now spreading around the world, is an expression of “raising” and “filial piety” in family relationships (20).

Influenced by the rapid spread and development of intergenerational learning concepts and practices around the world, the Chinese government, researchers, and practitioners have been actively engaged in the creation of learning families, learning societies, and learning nations in recent years. On October 23, 2021, China introduced the *Law of the People’s Republic of China on the Promotion of Family Education*, which emphasizes “mutual promotion and common growth among family members” (87). Subsequently, the construction of learning families with intergenerational learning programs under the cooperation of theorists and practitioners has been emerging all over China. It is important to note that the older adults who participated in this study were encouraged by Shuli to create a nationally influential intergenerational learning practice based on the context of home-school collaboration. Not only did they believe that intergenerational learning that took place within the home field was strongly endogenous and continuous, but they also highly recognized that intergenerational learning can help reduce the negative impact of the COVID-19 pandemic.

Yi Peng dared to explore new knowledge and skills. In his youth, he did various jobs such as construction, farming, and carving. He strived to integrate learning into his daily life and work and wanted to be a role model for his family as well as friends. It was with his belief in lifelong learning that he and other older adults interviewed responded positively to Shuli’s invitation to conduct an intergenerational learning program. As he expressed:

*Whereas family education or learning was previously characterized by the older adults teaching the younger how to behave, nowadays the older adults learning from the younger is becoming the norm in family relationships. The experience of learning tai chi and landscape painting from children and grandchildren during the COVID-19 pandemic made my daily life less dull. I think intergenerational learning will continue to be an important way for me to spend time with my children and grandchildren daily even after the COVID-19 pandemic is over.*

Ning Xiang, who like Yi Peng and other older adults was encouraged by the teacher of Shuli, did not hesitate to participate in the intergenerational learning program, which is an exploratory experiment in China. Like most of the older adults interviewed, the COVID-19 pandemic did not interrupt their lifelong learning habits. In addition, she repeatedly emphasized the short-term value of intergenerational learning as a successful response to the COVID-19 pandemic and the long-term significance of practicing lifelong learning. Her understanding of intergenerational learning programs is expressed as follows:

*Many older adults likely terminated their previously disciplined learning lives because of the COVID-19 pandemic. Fortunately, under the guidance of Shuli, I participated in the intergenerational learning program for my grandchildren and children. I think that intergenerational learning not only creates a positive mindset to face the COVID-19 pandemic but also opens up a new mode of lifelong learning other than going to an educational institution for older adults.*

As a mode of intergenerational learning that originated in developed Western countries, it possesses a unique social culture that is difficult to be imitated or replaced. While the emergence and development of intergenerational learning in China have benefited from the successful experiences of developed Western countries, it is also rooted in the ‘Chinese family culture’, which is characterized by the reciprocal relationship constructed by the concepts of ‘raising’ and ‘filial piety’. From the learning experiences of the older adults who participated in this study, it is clear that intergenerational learning is an important manifestation of learning from others that is different from self-directed learning, and an efficient shortcut for seeking survival rules and adapting to society in an uncertain society. Further, intergenerational learning is a readily available mode of informal learning to cope with the COVID-19 pandemic, which together with self-directed learning and institutionalized education institutions constitute the contemporary learning system for older adults in China.

#### 4.2.3 Offering online learning at home by school and society for older adults under the guidance of the government

With the promotion of UNESCO, the right to education, as a fundamental right of human beings, has been enshrined in the laws of many countries (88). *China’s Education Law* not only spells out that every person has the right to education but also emphasizes that the government must provide adequate and necessary conditions for the educated (89). In response to the challenges of an aging population and the construction of an educated nation, China issued a policy on the *Development Plan for Older Adults Education (2016–2020)* on October 5, 2016, which demonstrates that education for older adults is increasingly valued by the Chinese government (90). Reflected in practice is the rise of various education institutions, including multiple types of universities for older adults, community schools, open universities, and education or learning centers for older adults. Along with the iterative updating of global digital information technology, the digital transformation of education and learning for older adults has become an important direction for China’s education reform and development. In this regard, some studies proposed that the right to education in the artificial intelligence society should not be limited to face-to-face classroom teaching, but needs to be further expanded to non-face-to-face network online education and learning (91, 92).

With the aforementioned educational conceptualization and information technology support, the Chinese government enacted a series of policies to promote lifelong learning and geriatric rehabilitation, aiming to successfully address the challenges brought to older adults by the COVID-19 pandemic. For example, the Ministry of Civil Affairs of China, which was mentioned in the methodology, issued the *Notice on Strengthening Care Services for Older Adults with Special Difficulties During the Epidemic Prevention and Control Period* (60). Such top-down policies from the central to the local level help to ensure lifelong learning rights and opportunities for Chinese older adults, especially those with low social and cultural capital. All interviewees expressed that although the COVID-19 pandemic did hinder the normal operation of education for older adults, institutionalized curricula developed by universities and community schools for older adult under the guidance of the government ensured that they could live a life full of learning every day.

Before the COVID-19 pandemic, Xiu Yu insisted on going to the rural learning center on Mondays and Thursdays to participate in regular educational activities such as health care and intergenerational parenting. In response to home-based learning during the COVID-19 pandemic, the government set up an online learning platform for older adults throughout the administrative region by purchasing educational services from enterprises. She expressed this learning platform that satisfied her:

*I am grateful to the government for making great progress in developing education for older adults. Although I cannot go to the rural learning center for the time being, I can still study the educational resources recommended by the government at home through cell phone software. It is worth emphasizing that the government modified the fonts, speed of speech, and screen in the e-learning platform according to older adult’s physical and mental development characteristics.*

Like Xiu Yu and other older adults who participated in this study, Xia Qing was encouraged by Shuli and helped by her grandchildren to successfully participate in the e-learning platform constructed by Zhejiang Provincial University for Older Adults. This platform is an important experiment in digital transformation conducted by this institution. Before the COVID-19 pandemic, the platform had been created to promote the high-quality development of the education system for older adults. She is the first group of students on this online learning platform. The following were her experiences and lessons learned:

*I never thought I would have such a great opportunity to access high-quality educational resources. I have learned the correct exercise posture, high-profit financial management, and excellent photography skills from the electronic learning platform. Overall, this e-learning platform can meet my learning needs, and the convenient operating methods have brought me a good learning experience. I am very grateful to teacher Shu Li for recommending this high-quality learning platform to me through the intergenerational learning program.*

Numerous studies showed that digital transformation is an important force driving the development of education systems for older adults toward high quality around the world (76, 93). Under the combined influence of population aging, digital technology, and education concepts, China launched a huge project for digital transformation. The practice has already proved that systematic courses guided by the government and created by enterprises and education institutions have helped older adults maintain their enthusiasm and motivation for learning during the COVID-19 pandemic. While the older adults who participated in this study may have faced difficulties such as digital technology in the process of e-learning, self-directed learning as well as intergenerational learning in the family collectively empowered China’s education for older adults to move toward quality development.

## 5 Discussion and conclusion

The sudden COVID-19 pandemic has had a tremendous and profound impact on the world’s economy, politics, culture, ecology, and education (94). The physical and mental development of older adults, who are the typical representatives of socially disadvantaged groups, has been seriously harmed by this uncontrollable and immeasurable social crisis (13, 14). This study explored the learning challenges of the COVID-19 pandemic to older adults with low social and cultural capital in rural eastern China, and how they successfully coped with these uncertain challenges from personal initiative, family help, and social support. This study took home and school cooperation in China as the background and conducted in-depth interviews with 12 older adults with the help of Shuli from a rural school in Zhejiang province. This study found that the COVID-19 pandemic brought at least two more prominent challenges to older adult learning. On the one hand, in the face of the COVID-19 pandemic, China formulated a policy of working, studying, and living at home to prevent older adults from going to educational institutions outside school. This undoubtedly further affects the right of older adults to education and learning opportunities. On the other hand, the COVID-19 pandemic accelerated the pace of society entering the digital era. Older adults who have lived in traditional industrialization for a long time find it almost difficult to survive in a society full of information, numbers, and technology. With education shifts from traditional models to digital forms, older adults often face challenges such as a lack of knowledge, inadequate skills, and psychological setbacks. This study further found that older adults were not forced to accept these challenges passively and helplessly, but created lifelong learning practices with Chinese characteristics, connecting individuals, families, and society through internal redemption and external help. Firstly, based on the subjective initiative of older adults, they tended to construct self-directed learning methods through television, mobile phones, and tablets. Secondly, under the influence of Chinese family culture characterized by “raising” and “filial piety,” they created intergenerational learning activities in the family. Third, the e-learning platform developed by enterprises and schools for older adults under the guidance of the government provided them with an important guarantee and strength to maintain lifelong learning.

In the literature review section, I already presented in detail that existing studies on the impact of the COVID-19 pandemic on older adults focused more on physical rehabilitation and psychological counseling (26, 30), relatively neglecting the value and significance of lifelong education and learning for coping with the social crisis (16, 17, 24). Some studies in the field of pedagogy began to pay more attention to the right to education of older adults, especially during the COVID-19 pandemic, there was a strong demand for learning, and further provided practitioners with necessary and even rich educational resources and learning opportunities (31, 35, 38). However, these studies on educational rights and learning resources mainly focused on older adults with high social and cultural capital in the urban of developed countries and relatively ignored the older adults with low social and cultural capital in rural developing countries to some extent (49, 54). Obviously, the current research horizons and results on the challenges and coping methods of the COVID-19 pandemic to older adults were not broad enough from the perspective of the human destiny community (95). Therefore, to further explore and disseminate the theoretical exploration, policy support, and practical experience of how to provide educational resources and learning opportunities for the rural disadvantaged older adults in developing countries during or after the COVID-19 pandemic (49), this study as a typical case attempted to take the successful and effective practice of lifelong education and learning of the rural older adults with low social and cultural capital in eastern China. By analyzing the learning challenges and solutions faced by older adults with low social and cultural capital, this study helped to make up for the deficiency of the existing literature which tended to discuss the lifelong learning of older adults with high social and cultural capital rather than older adults with low social and cultural capital in developed countries. In addition, this study further provided wisdom and solutions with Chinese characteristics for building a more inclusive, fair, and high-quality education and support service system for older adults in the sense of a community with a shared future of mankind.

This study further emphasized that in the dialog and discussion with existing literature, in addition to the two challenges mentioned above and the theoretical value of the Chinese education system for older adults constructed by individuals, families, and society, this study enlightened significance for policy formulation and practical reform of lifelong education and learning. Policymakers should maximize the scope of influence of the policy, and strive to ensure that the position, content, and implementation of the policy take into account the different social and cultural capital of older adults so that all older adults can practice the concept of lifelong learning in their interactions with family and society. In addition, practitioners engaged in education for older adults should adhere to the humanism of inclusiveness for all, establish intergenerational learning, friendly schools for older adults, and online education, as well as help each older adult to choose their own educational model and learning methods. In addition, at a time when population aging is intensifying, education digital transformation is fully launched, and education for older adults is more important, actively building a harmonious society and promoting human beings to move toward fairness and justice are important issues that countries around the world must think deeply about.

Although this study on the learning of rural older adults in some areas of China during the COVID-19 pandemic from a global perspective has some inevitable limitations, such as small sample size and cultural differences in some countries, this study is in line with the existing social culture, information technology and education for older adults. Future research may continue to expand to sample older adults in central and western China and attempt to invite them to critically reflect on the current Chinese education service system for older adults, especially when studying their strategies for building education for older adults. This not only opens up a possible way for older adults to improve their self-reflection awareness and self-improvement ability, but also provides richer possibilities for researchers to challenge their dominant ideas in education for older adults, cultural differences, social capital, and information technology.

## Data Availability

The original contributions presented in the study are included in the article/supplementary material, further inquiries can be directed to the corresponding author.
